# Supplementary material for: Biological Aging Measures Based on Blood DNA Methylation and Risk of Cancer: A Prospective Study
Source: JNCI Cancer Spectr. 2020 Nov 16;5(1):pkaa109. doi: 10.1093/jncics/pkaa109 (PMC7791618; doi:10.1093/jncics/pkaa109)
Supplement: pkaa109_Supplementary_Data [file pkaa109_supplementary_data.pdf]

SUPPLEMENTARY MATERIALS

Biological aging measures based on DNA methylation and risk of cancer: a prospective study

Dugué PA et al., Email: pierre-antoine.dugue@monash.edu

Precision Medicine, School of Clinical Sciences at Monash Health, Monash University, VIC, Australia.

SUPPLEMENTARY METHODS

DNA extraction and bisulfite conversion, and DNA methylation data processing

Bisulphite conversion of DNA and Illumina Infinium methylation assay

Genomic DNA was extracted from mononuclear cells using the QIAamp 96 DNA blood kit (Qiagen), and from Guthrie card samples as previously described **[1].** Briefly, twenty blood spots of 3.2 mm diameter were punched from the Guthrie card and lysed in phosphate buffered saline using TissueLyser (Qiagen). The resulting supernatant was processed using Qiagen mini spin columns according to the manufacturer's protocol. The quality and quantity of DNA was assessed using the Quant-iT™ Picogreen® dsDNA assay measured on the Qubit® Fluorometer (Life Technologies, Grand Island, NY), with a minimum of 0.3 µg DNA considered acceptable for methylation analysis. Genomic DNA was bisulphite converted using the EZ DNA Methylation-Gold kit (Zymo Research, Irvine, CA) following the manufacturer's instructions. The Illumina Infinium HumanMethylation450K BeadChip (HM450K) array (Illumina, Inc.; San Diego, CA, USA) was used to measure DNA methylation. This array covers 99% of the RefSeq genes and uses a combination of two distinct probe types (Infinium I and II) to detect the methylation status of 485,577 CpGs in the human genome at a single base resolution **[2].** Samples in each nested case-control study and the longitudinal study were assayed at non-overlapping time periods. In each study, samples were randomly assigned to chips and processed as per the Illumina protocol. All laboratory work, including DNA extraction, bisulphite conversion and Infinium assaying was performed at the Genetic Epidemiology Laboratory, University of Melbourne.

Normalization and quality control of methylation data

The same normalization and quality control procedures were applied to methylation data in all eight studies (case-control and longitudinal). Raw .IDAT files were imported into R using the *minfi* package **[3].** Illumina's background correction was applied based on internal control probes. Subset-quantile within-array normalisation (SWAN) was used to correct for the technical variability between the two probe types of the HM450K array **[4]**. The 'getSex' function of the *minfi* package **[3]** was used to predict sex for each sample. Samples for which predicted sex was inconsistent with recorded sex were excluded from further analyses. For each sample, each CpG with a detection P value >0.01 was assigned as missing. Samples with missing values for more than 5% of probes were excluded. CpG sites were excluded if they were missing for more than 20% of samples. β-values, which range from zero to one and correspond to the percentage of methylation, were calculated for each CpG using *minfi*. β-values were transformed into M-values using the formula: $M = log_2(\frac{\beta}{1-\beta})$. **[5]**.

REFERENCES

1. Joo JE, Wong EM, Baglietto L, *et al.* The use of DNA from archival dried blood spots with the Infinium HumanMethylation450 array. BMC Biotechnol 2013;13:23.

2. Bibikova M, Barnes B, Tsan C, *et al.* High density DNA methylation array with single CpG site resolution. Genomics 2011;98(4):288-95.

3. Aryee MJ, Jaffe AE, Corrada-Bravo H, *et al.* Minfi: a flexible and comprehensive Bioconductor package for the analysis of Infinium DNA methylation microarrays. Bioinformatics 2014;30(10):1363-9.

4. Maksimovic J, Gordon L, Oshlack A. SWAN: Subset-quantile within array normalization for illumina infinium HumanMethylation450 BeadChips. Genome Biol 2012;13(6):R44.

5. Du P, Zhang X, Huang CC, *et al.* Comparison of Beta-value and M-value methods for quantifying methylation levels by microarray analysis. BMC Bioinformatics 2010;11:587.

6. Dugué PA, Bassett JK, Joo JE, *et al.* DNA methylation-based biological aging and cancer risk and survival: Pooled analysis of seven prospective studies. Int J Cancer 2018;142(8):1611-1619.

Supplementary Figure 1. Exclusion of outliers based on the distribution of age-adjusted measures of biological aging (all participants), using 5 standard deviations as cut-off. Age-adjusted measures have mean=0 and variance=1. Six participants were identified as outliers and the corresponding case-control pairs were excluded

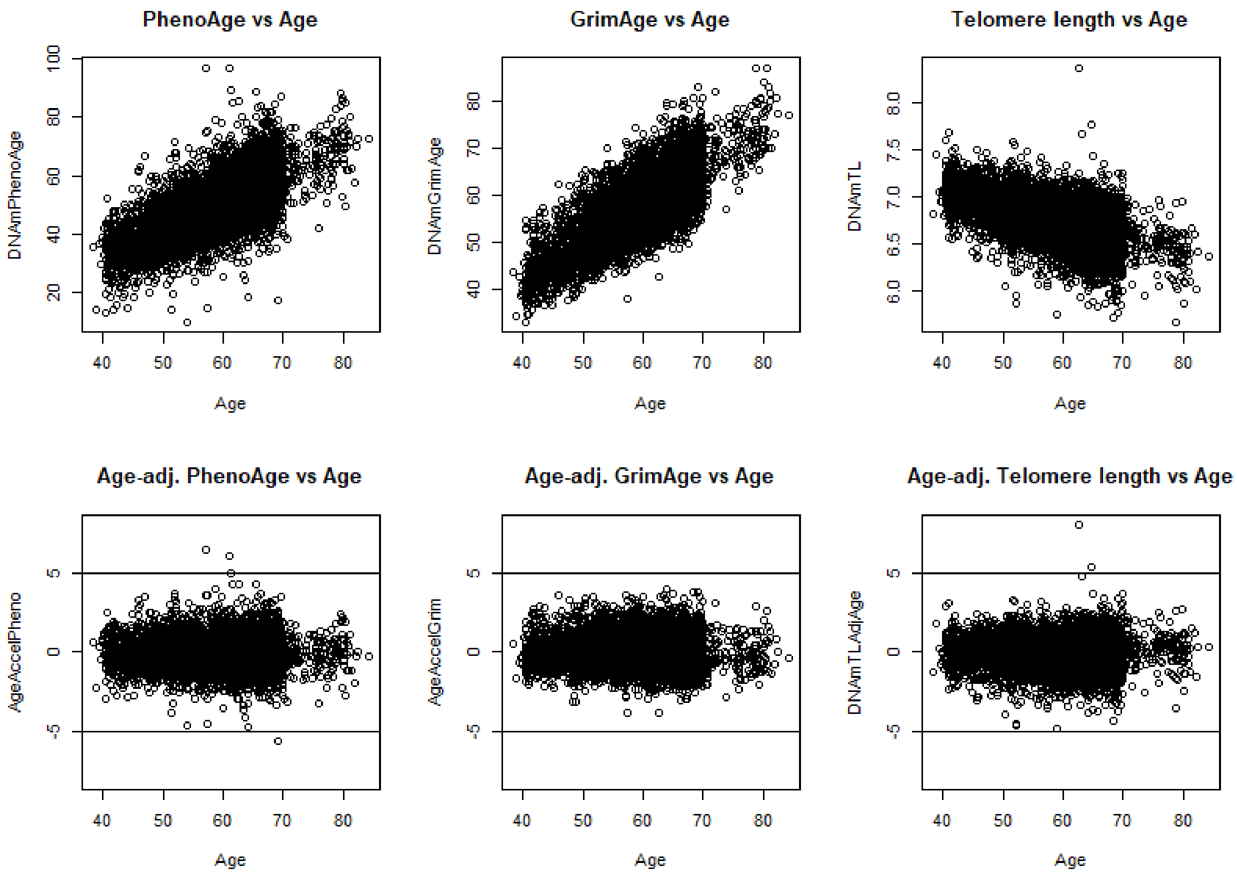

Supplementary Table 1. Pearson correlations between methylation-based predictors and chronological age

	Chronological age	PhenoAge	GrimAge	Telomere length	Age-adjusted PhenoAge	Age-adjusted GrimAge
**PhenoAge**	0.70					
**GrimAge**	0.80	0.71				
**Telomere length**	-0.55	-0.54	-0.60			
**Age-adjusted PhenoAge**	0.00	0.71	0.19	-0.21		
**Age-adjusted GrimAge**	0.00	0.24	0.57	-0.24	0.34	
**Age-adjusted telomere length**	0.00	-0.18	-0.17	0.83	-0.25	-0.29

Supplementary Table 2. Pearson correlations between three age-adjusted methylation measures of biological aging included in this study, and five age-adjusted first-generation measures presented in [6]

	AA-Horvath	AA-Hannum	IEAA-Horvath	IEAA-Hannum	EEAA
**Age-adjusted PhenoAge**	0.35	0.39	0.35	0.29	0.41
**Age-adjusted GrimAge**	0.12	0.26	0.13	0.19	0.28
**Age-adjusted telomere length**	-0.25	-0.42	-0.11	-0.22	-0.50

AA-Horvath: Age acceleration from the Horvath clock; AA-Hannum; Age acceleration from the Hannum clock; IEAA-Horvath: intrinsic epigenetic age acceleration from the Horvath clock; IEAA-Hannum: intrinsic epigenetic age acceleration from the Horvath clock; EEAA: enhanced Hannum age acceleration

Supplementary Table 3. Association (Rate Ratios (RR), 95% confidence intervals (95% CI) between three methylation-based measures of aging and cancer risk in the Melbourne Collaborative Cohort Study, by cancer subtypes.

Cancer type	Aging measure		RR	95% CI	P	Heterogeneity P (Model 2)	
**Colorectal cancer**							
**Colon (N=525)**	PhenoAge	Model 1	1.17	1.03-1.33	0.018		
		Model 2	1.16	1.01-1.32	0.029		
	GrimAge	Model 1	1.15	1.00-1.31	0.043		
		Model 2	1.16	0.98-1.38	0.088		**P**
	Telomere length	Model 1	0.99	0.86-1.12	0.832	PhenoAge	0.16
		Model 2	1.01	0.88-1.16	0.863	GrimAge	0.51
**Rectum (N=288)**	PhenoAge	Model 1	1.34	1.11-1.62	0.003	Telomere length	0.74
		Model 2	1.38	1.12-1.69	0.003		
	GrimAge	Model 1	1.32	1.08-1.60	0.006		
		Model 2	1.29	1.00-1.66	0.048		
	Telomere length	Model 1	0.97	0.82-1.15	0.695		
		Model 2	0.97	0.81-1.17	0.779		
**B-cell mature neoplasm**							
**Multiple myeloma (N=108)**	PhenoAge	Model 1	1.02	0.76-1.36	0.912		
		Model 2	1.15	0.82-1.60	0.425		
	GrimAge	Model 1	1.02	0.74-1.41	0.901		
		Model 2	0.96	0.62-1.50	0.870		
	Telomere length	Model 1	0.88	0.67-1.15	0.356		
		Model 2	0.84	0.62-1.14	0.254		
**Follicular lymphoma (N=78)**	PhenoAge	Model 1	1.22	0.87-1.72	0.243		
		Model 2	1.32	0.87-2.00	0.187		
	GrimAge	Model 1	1.02	0.68-1.53	0.924		
		Model 2	1.11	0.63-1.93	0.722		**P**
	Telomere length	Model 1	0.73	0.53-1.02	0.069	PhenoAge	0.05
		Model 2	0.72	0.48-1.08	0.116	GrimAge	0.52
**Low-grade NHL (N=131)**	PhenoAge	Model 1	1.72	1.28-2.29	0.000	Telomere length	0.16
		Model 2	1.90	1.37-2.62	0.000		
	GrimAge	Model 1	0.98	0.75-1.27	0.865		
		Model 2	1.14	0.79-1.63	0.485		
	Telomere length	Model 1	1.15	0.91-1.46	0.235		

		Model 2	1.15	0.88-1.49	0.312		
**High-grade NHL (N=106)**	PhenoAge	Model 1	1.02	0.78-1.34	0.876		
		Model 2	1.08	0.80-1.45	0.607		
	GrimAge	Model 1	0.77	0.55-1.10	0.152		
		Model 2	0.76	0.49-1.17	0.208		
	Telomere length	Model 1	0.85	0.66-1.10	0.230		
		Model 2	0.82	0.62-1.08	0.159		
**Prostate cancer**							
**Prostate: aggressive (N=417)**	PhenoAge	Model 1	1.03	0.90-1.19	0.806		
		Model 2	1.04	0.89-1.20	0.815		
	GrimAge	Model 1	0.95	0.81-1.10	0.458		
		Model 2	0.97	0.79-1.17	0.702		**P**
	Telomere length	Model 1	1.04	0.89-1.22	0.499	PhenoAge	0.35
		Model 2	1.04	0.89-1.23	0.494	GrimAge	0.16
**Prostate: non-aggressive (N=429)**	PhenoAge	Model 1	0.92	0.80-1.07	0.287	Telomere length	0.91
		Model 2	0.94	0.80-1.09	0.382		
	GrimAge	Model 1	0.81	0.69-0.95	0.012		
		Model 2	0.79	0.64-0.97	0.026		
	Telomere length	Model 1	1.08	0.93-1.26	0.310		
		Model 2	1.06	0.90-1.24	0.481		
**Urothelial cancer**							
**Urothelial: invasive (N=182)**	PhenoAge	Model 1	1.21	0.97-1.50	0.087		
		Model 2	1.14	0.90-1.45	0.275		
	GrimAge	Model 1	1.50	1.19-1.89	0.001		
		Model 2	1.15	0.85-1.57	0.361		
	Telomere length	Model 1	0.87	0.70-1.08	0.205		**P**
		Model 2	0.94	0.74-1.20	0.626	PhenoAge	0.83
**Urothelial: superficial (N=222)**	PhenoAge	Model 1	1.21	0.99-1.48	0.057	GrimAge	0.60
		Model 2	1.18	0.96-1.46	0.120	Telomere length	0.94
	GrimAge	Model 1	1.30	1.07-1.59	0.010		
		Model 2	1.29	0.99-1.68	0.063		
	Telomere length	Model 1	0.92	0.76-1.12	0.393		
		Model 2	0.95	0.77-1.18	0.667		

Supplementary Table 4. Association (Rate Ratios (RR), 95% confidence intervals (95% CI) between *PhenoAge* and *GrimAge* (per 5 years) and cancer risk in the Melbourne Collaborative Cohort Study

			PhenoAge			*GrimAge*		
**Cancer type**	**N cases**		**RR**	**95% CI**	**P**	**RR**	**95% CI**	**P**
**Colorectal cancer**	814	Model 1	1.13	1.06-1.21	0.0005	1.22	1.08-1.39	0.001
		Model 2	1.13	1.05-1.21	0.0008	1.21	1.04-1.42	0.02
**Gastric cancer**	166	Model 1	0.96	0.84-1.11	0.60	1.03	0.81-1.30	0.84
		Model 2	0.97	0.83-1.13	0.69	1.04	0.75-1.45	0.81
**Kidney cancer**	139	Model 1	1.16	0.98-1.38	0.09	1.31	0.97-1.76	0.07
		Model 2	1.18	0.96-1.45	0.12	1.36	0.89-2.08	0.15
**Lung cancer**	327	Model 1	1.15	1.04-1.27	0.007	1.95	1.52-2.51	$1x10^{-7}$
		Model 2	1.15	1.03-1.28	0.011	1.97	1.51-2.56	$6x10^{-7}$
**Mature B-cell neoplasms**	426	Model 1	1.14	1.05-1.25	0.003	0.93	0.78-1.11	0.45
		Model 2	1.16	1.05-1.27	0.002	0.94	0.75-1.17	0.56
**Prostate cancer**	847	Model 1	0.98	0.92-1.05	0.58	0.86	0.76-0.98	0.02
		Model 2	0.99	0.92-1.06	0.74	0.86	0.74-1.01	0.07
**Urothelial cancers**	404	Model 1	1.13	1.03-1.25	0.01	1.45	1.22-1.72	$2x10^{-5}$
		Model 2	1.11	1.00-1.22	0.05	1.25	1.00-1.55	0.05
**All types**	3,086	Model 1	1.09	1.05-1.12	$3x10^{-6}$	1.14	1.07-1.21	$5x10^{-5}$
		Model 2	1.08	1.04-1.12	$9x10^{-6}$	1.13	1.05-1.22	0.002

Model 1: No adjustment other than that provided by the matching variables (age, sex, country of birth (Australia / Northern Europe / Southern Europe), sample type (peripheral blood mononuclear cells / dried blood spots / buffy coats); lung cancer study: additional matching for smoking status (never; former, quitting <10 years; former, quitting ≥10 years; current, smoking <15 cigarettes/day; current smoking ≥15 cigarettes/day)

Model 2: Additional adjustment for smoking (current / former / never), smoking pack-years, age at starting smoking (4 categories), time since quitting smoking (4 categories), BMI (in kg/m^2), height (in m), alcohol consumption (in grams/day), physical activity (categorised score), dietary quality (Alternative Healthy Eating Index 2010, Chiuve et al., J Nutr, 2012), socioeconomic status score (at the local area level, ranging from 1 to 10), education score (ordinal variable ranging from 1: “primary school” to 8:“postgraduate degree”)

Supplementary Table 5. Association (Rate Ratios (RR), 95% confidence intervals (95% CI) between five first-generation methylation-based measures of aging (per one standard deviation) and cancer risk in the Melbourne Collaborative Cohort Study

			AA-Horvath			*AA-Hannum*			*IEAA-Horvath*			*IEAA-Hannum*			*EEAA*		
**Cancer type**	**N cases**		**RR**	**95% CI**	**P**	**RR**	**95% CI**	**P**	**RR**	**95% CI**	**P**	**RR**	**95% CI**	**P**	**RR**	**95% CI**	**P**
**Colorectal cancer**	814	Model 1	1.11	1.00-1.24	0.05	1.11	0.99-1.23	0.06	1.10	0.99-1.23	0.08	1.06	0.95-1.17	0.32	1.10	0.99-1.23	0.07
		Model 2	1.10	0.99-1.23	0.09	1.10	0.99-1.23	0.08	1.08	0.97-1.21	0.16	1.04	0.94-1.16	0.44	1.10	0.99-1.23	0.09
**Gastric cancer**	166	Model 1	0.90	0.71-1.13	0.36	1.07	0.85-1.34	0.58	1.00	0.80-1.26	0.98	1.16	0.92-1.45	0.21	1.06	0.85-1.32	0.61
		Model 2	0.94	0.73-1.21	0.64	1.14	0.89-1.48	0.30	1.04	0.81-1.34	0.75	1.24	0.96-1.59	0.10	1.12	0.87-1.44	0.38
**Kidney cancer**	139	Model 1	1.13	0.86-1.49	0.39	1.46	1.11-1.92	0.01	1.11	0.86-1.44	0.43	1.42	1.07-1.88	0.01	1.44	1.11-1.86	0.01
		Model 2	1.20	0.87-1.66	0.27	1.61	1.14-2.27	0.01	1.15	0.86-1.55	0.35	1.52	1.07-2.16	0.02	1.55	1.12-2.15	0.01
**Lung cancer**	327	Model 1	0.99	0.85-1.16	0.91	1.09	0.93-1.29	0.29	0.96	0.83-1.11	0.60	1.04	0.89-1.21	0.64	1.14	0.97-1.35	0.12
		Model 2	1.03	0.87-1.22	0.71	1.09	0.92-1.30	0.31	1.00	0.85-1.17	0.99	1.04	0.89-1.23	0.60	1.15	0.96-1.37	0.12
**Mature B-cell neoplasms**	426	Model 1	1.22	1.07-1.38	<0.01	1.24	1.09-1.41	<0.01	1.03	0.91-1.17	0.63	1.01	0.89-1.15	0.84	1.32	1.15-1.5	<0.01
		Model 2	1.22	1.07-1.39	<0.01	1.25	1.10-1.43	<0.01	1.03	0.90-1.17	0.68	1.01	0.89-1.16	0.85	1.33	1.16-1.53	<0.01
**Prostate cancer**	847	Model 1	1.04	0.93-1.15	0.51	0.95	0.85-1.06	0.38	1.05	0.95-1.16	0.37	0.97	0.87-1.09	0.63	0.95	0.85-1.05	0.31
		Model 2	1.02	0.92-1.14	0.65	0.95	0.85-1.06	0.37	1.04	0.93-1.15	0.51	0.97	0.87-1.09	0.60	0.95	0.85-1.06	0.34
**Urothelial cancers**	404	Model 1	1.02	0.88-1.18	0.80	1.06	0.92-1.22	0.45	1.01	0.88-1.16	0.90	1.00	0.87-1.15	1	1.09	0.95-1.26	0.23
		Model 2	1.03	0.89-1.20	0.66	1.05	0.90-1.22	0.56	1.03	0.89-1.19	0.74	1.00	0.87-1.17	0.95	1.07	0.92-1.25	0.39
**All types**	3,086	Model 1	1.07	1.02-1.13	0.01	1.09	1.04-1.15	<0.01	1.04	0.99-1.10	0.13	1.04	0.98-1.09	0.19	1.11	1.05-1.17	<0.01
		Model 2	1.07	1.01-1.13	0.01	1.09	1.04-1.15	<0.01	1.04	0.98-1.09	0.19	1.03	0.98-1.09	0.24	1.11	1.05-1.17	<0.01

Model 1: No adjustment other than that provided by the matching variables (age, sex, country of birth (Australia / Northern Europe / Southern Europe), sample type (peripheral blood mononuclear cells / dried blood spots / buffy coats); lung cancer study: additional matching for smoking status (never; former, quitting <10 years; former, quitting ≥10 years; current, smoking <15 cigarettes/day; current smoking ≥15 cigarettes/day)
Model 2: Additional adjustment for smoking (current / former / never), smoking pack-years, age at starting smoking (4 categories), time since quitting smoking (4 categories), BMI (in kg/m^2), height (in m), alcohol consumption (in grams/day), physical activity (categorised score), dietary quality (Alternative Healthy Eating Index 2010, Chiuve et al., J Nutr, 2012), socioeconomic status score (at the local area level, ranging from 1 to 10), education score (ordinal variable ranging from 1: "primary school" to 8:"postgraduate degree")
AA-Horvath: Age acceleration from the Horvath clock; AA-Hannum; Age acceleration from the Hannum clock; IEAA-Horvath: intrinsic epigenetic age acceleration from the Horvath clock; IEAA-Hannum: intrinsic epigenetic age acceleration from the Horvath clock; EEAA: enhanced Hannum age acceleration
